# Experimental Evidence for the Pore Size Dependence of Elastic Properties in a Liquid Adsorbate Confined to Nanopores

**DOI:** 10.1021/acs.jpclett.5c03903

**Published:** 2026-01-28

**Authors:** Klaus Schappert, Rolf Pelster

**Affiliations:** FR Physik, Universität des Saarlandes, Campus E2 6, 66123 Saarbrücken, Germany

## Abstract

The elasticity of adsorbates is relevant for many applications of porous materials. Theoretical studies predict a pronounced increase of elastic moduli for adsorbates under nanoconfinement, but an experimental confirmation is still missing. Here we present an ultrasonic study on the longitudinal modulus β_*Ar*,*ads*_ of liquid argon in porous glass samples with different pore radii between 1.8 and 12.8 nm. The analysis of the measured moduli of empty, β_0_, and filled samples, β, reveals that the modulus of the adsorbate, β_*Ar*,*ads*_, increases linearly with the inverse pore radius, 1/*r*_*P*_, as predicted by theory.

## Graphical abstract

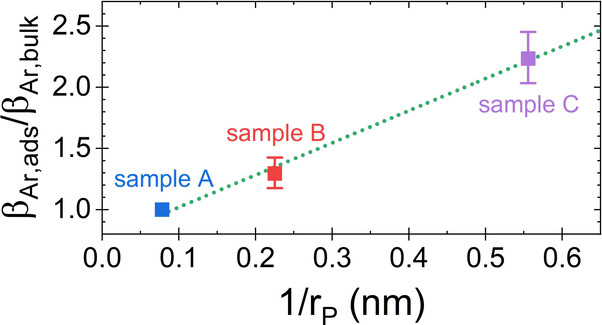

Many materials—both natural and synthesized—have a porous structure. Examples range from sandstones and porous rocks to porous glasses, aluminum, silicon, and metal–organic frameworks. In nature, the pores are generally filled with fluids like water, oil or gases. Porous media can be used for numerous applications including filtration and separation of fluids or also as a storage medium for carbon dioxide, methane, hydrogen, drugs, and other substances.^1–12^ Consequently, porous materials are of great relevance in many fields (e.g., petroleum engineering, chemical, pharmaceutical and automobile industry).^1–12^ Generally, properties of substances confined in pores can deviate from bulk properties, notably for nanopores (pore diameter *d*_*P*_ < 100 nm).^9,13^ Thus, theoretical and experimental studies revealed deviations of the physical behavior of adsorbates (in comparison to the bulk state) and investigated the impact of adsorption on the matrix itself.^1,9,13–17^ The extent of the observed effects are in particular related to the pore size, the structure of the pore surface and the interaction between matrix and adsorbate.^9,13,14,16,18–20^

For many of the above-mentioned research areas the elasticity of porous media is very significant, which has resulted in a multitude of studies in this field of research.^1,18–52^ Adsorption in pores is related to a so-called solvation pressure that is exerted on the porous material, which causes the well-known effect of sorption-induced deformation.^14,33,53–58^ The extent of this deformation depends in turn on the elastic properties of the porous material.^14,15,31,59–61^ But it is also obvious that adsorption changes the effective elastic behavior of porous structures.^37,45,46,51^ Generally, more force has to be applied for the deformation of a filled porous medium than for the empty medium, i.e., the effective modulus of the filled medium, *M*, is higher than that of the empty material, *M*_0_ (with *M* being either the bulk modulus *K*, the longitudinal modulus β, or the shear modulus *G*).^45,46,51^ Of course, the difference between the modulus *M* and that of the empty sample, *M*_0_, depends decisively on the elasticity of the adsorbate, *M*_*ads*_. The exact relation between measured effective quantities and intrinsic elastic properties of an adsorbate can differ and usually its determination requires an effective medium analysis.^39^

Previous research showed that adsorption influences the elastic properties of the nanoconfined adsorbate.^1^ Thus, experiments revealed that the curvature of liquid–vapor interfaces at the pore ends changes the elasticity of the adsorbate.^45,51^ This experimentally observed effect, which was confirmed by theory, is a result of the Laplace pressure that is related to the curvature of these menisci.^35,45^ The Laplace pressure is part of the solvation pressure, which also causes a deformation of porous materials (sorption-induced deformation).^14^ Experimental and theoretical studies also showed that even at the saturation vapor pressure, the modulus of an adsorbate differs from that of the bulk material^1,18–20,35,41,44^ and the extent of the enhancement of the modulus varies for different adsorbates.^18–20,41,44^ Obviously, for a given pore size the strength of the interaction between adsorbate and pore surface influences the elasticity of the adsorbate decisively.^1,18–20,41,44,62^ Thus, the enhancement of the elastic moduli of confined nitrogen or oxygen is much more pronounced than for argon.^18–20,41,44^

Gennady Y. Gor and his group discovered a linear dependence between the bulk modulus of confined liquid adsorbates (argon, nitrogen, methane) and the inverse pore size (note that for liquids the bulk modulus *K*_*ads*_ equals the longitudinal modulus β_*ads*_).^1,22,30,32,34,35,63^ This linear dependence of an adsorbate’s modulus on the inverse pore size can be attributed to the variation of the solvation pressure with the pore size:^35^ In fully saturated pores the sorption-induced change of pressure can be written in terms of macroscopic variables as −γ_*SL*_/*r*_*P*_ with γ_*SL*_ being the surface tension between the solid pore walls and the liquid adsorbate. Since the bulk modulus of a liquid increases (approximately) linearly as a function of pressure, also the enhancement of *K*_*ads*_ is expected to be proportional to the inverse pore radius.^1,40,43^ Of course, such a macroscopic description does not exclude phenomena on a microscopic scale such as fluid-layering at the pore walls.^35^

For their theoretical studies Gor et al. used simulations and DFT (density functional theory) calculations.^1,22,30,32,34,35,63^ Most of these simulations were performed for spherical pores with a (smooth) silica surface, however, Dobrzanski et al. found also for argon in smooth cylindrical pores a linear dependence, though it is slightly weaker.^1,30,35^ Their findings indicate that in small nanopores (pore size in the range of 2 nm) the adsorbate’s modulus deviates mostly by a factor of about two from the modulus of the bulk substance (for temperatures near the normal boiling point of the adsorbates).^30,32,34,35,63^ Such a strong enhancement was found for both the weakly interacting adsorbate argon and for nitrogen.^32,34,35,63^ But obviously the strength of the enhancement depends not only on pore geometry but also significantly on temperature (cp. data shown in ref 30). Thus, for higher temperatures the modulus of argon in cylindrical nanopores with a pore size of 2 nm is predicted to be even four times as high as that for bulk argon at the same temperature (cp. data in Figure 11 in ref 30). Of course, for large pores the elastic properties of an adsorbate are expected to be equal to those of the bulk liquid (see, e.g., ref 30 for argon). Accordingly, also for confined methane a recent molecular simulation study showed that in large pores the adsorbate’s modulus approaches the bulk value.^22^

Experimentally the theoretically predicted pore size dependence has previously not been investigated. The performance of the required experiments is rather challenging and as noted by Dobrzanski et al.^1^ there is a demand for more measurements. Experimental issues can make the determination of effective moduli of (filled or empty) porous samples a difficult task. In particular, the determination of changes of the effective modulus in consequence of adsorption requires a very high accuracy for the determination of ultrasonic velocities. Besides, the quality of the samples (e.g., no cracks, polished surfaces) and also the availability of samples with varying pore sizes have previously hindered the experimental research of the pore size dependence of an adsorbate’s modulus.

Here we have studied the longitudinal modulus β_*Ar*,*ads*_ of liquid argon confined to the pores of nanoporous glasses with different pore sizes. The experimental research of the influence of the pore size on the elastic properties of the adsorbate requires samples with different pore sizes but with a similar chemical composition. In particular, the chemical compounds at the pore surface should be the same as a variation of the pore surface might result in a variation of the interaction strength with the adsorbate and hence of the adsorbate’s elasticity.^18–20,64^ For this study we have used three different nanoporous glass monoliths with interconnected pores and uniform porosity.^65,66^ The samples were produced by Particle Solutions, LLC (Florida, USA) using a sol–gel technique.^65–67^ The pore size distributions, which we have determined from argon isotherms at 86 K (see Figure 1a), show a significant variation of the pore radii of the three samples (see Figure 1b). The respective size distributions exhibit a maximum at *r*_*P*_ = 12.8 nm (sample A), *r*_*P*_ = 4.4 nm (sample B), and *r*_*P*_ = 1.8 nm (sample C).

**Figure 1:**
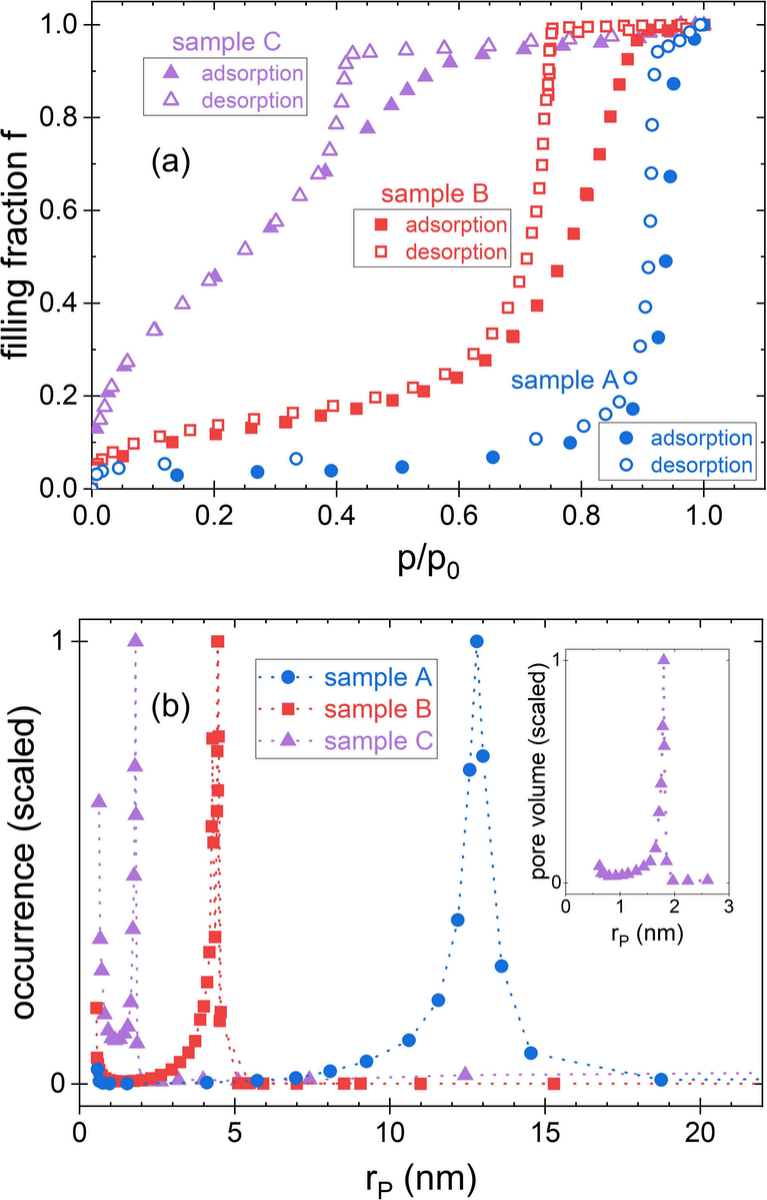
(a) Argon sorption isotherms of the three porous glass monoliths studied (measured at 86 K). The filling fraction of the pores (*f* = *n*/*n*_0_, with the molar amount of argon, *n*, and its maximum, *n*_0_) is shown as a function of the relative pressure *p*/*p*_0_ (with the saturation vapor pressure *p*_0_). (b) Pore size distributions of the samples determined using the desorption branch of the isotherms. The figure shows the scaled occurrence of the radii. The pore radii with the highest occurrence are: sample A: *r*_*P*_ = 12.8 nm, sample B: *r*_*P*_ = 4.4 nm, and sample C: *r*_*P*_ = 1.8 nm. Note that the smaller second peak in the occurrence that is noticeable for very small pore radii (at *r*_*P*_ ≈ 0.5 nm in sample C) corresponds to a negligible pore volume. The inset shows the scaled pore volume of the radii for sample C.

In addition, the porosity of the samples, i.e., the pore volume per sample volume ϕ = *V*_*pores*_/*V*_*sample*_, varies considerably between ≈35% and ≈60% (see Table 1). This becomes noticeable in the effective density ρ_0_ of the three samples. Accordingly, Figure 2a reveals that the effective density ρ_0_ decreases continuously – roughly linearly – with increasing porosity ϕ.

**Figure 2:**
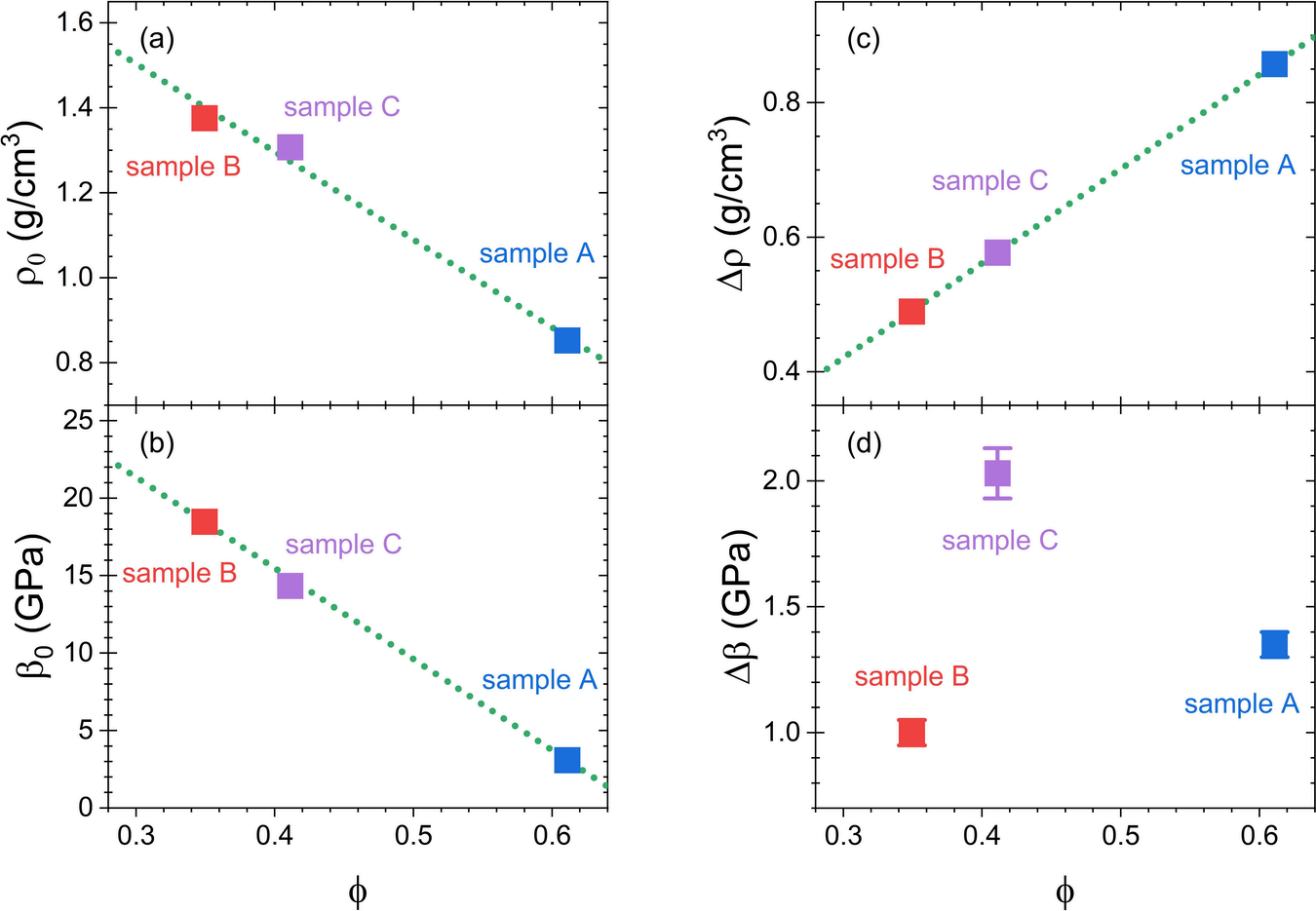
(a) Dependence of the effective density ρ_0_ of the empty porous glass monoliths on the porosity ϕ. The density ρ_0_ decreases continuously with increasing porosity ϕ. (b) Effective longitudinal modulus β_0_ of the empty sample as a function of the porosity ϕ at 86 K. The experimentally determined modulus of the empty sample exhibits an almost linear dependence on the porosity ϕ, i.e., it holds β_0_ = β_*m*,0_ – *aϕ* with the extrapolated modulus of the matrix material, β_*m*,0_. A linear fit to the data for β_0_ yields β_*m*,0_ = 38.97 ± 0.28 GPa and *a* = 58.73 ± 0.47 GPa. (c) The contribution Δρ = ρ – ρ_0_ of the adsorbed argon to the effective density increases linearly with porosity. (d) The contribution of the adsorbate to the effective longitudinal modulus, Δβ = β – β_0_, shows no systematic dependence on the porosity (in particular, no linear increase with ϕ), which suggests that β_*Ar*,*ads*_ varies for the different samples (see text). The dotted lines in (a)–(c) are linear fits to the data.

**Table 1:** Properties of the Nanoporous Glass Samples Studied^a^

	sample		
	A	B	C
pore radius r_*P*_ (nm)	12.8	4.4	1.8
porosity ϕ	0.611 ± 0.001	0.349 ± 0.001	0.411 ± 0.001
**empty sample:**			
density ρ_0_ (kg/m^3^)	852	1376	1307
velocity **c*_*l*,0_* (m/s)	1903.8 ± 1.4	3664.5 ± 1.6	3311.1 ± 7.7
modulus β_0_ (GPa)	3.09 ± 0.01	18.48 ± 0.02	14.33 ± 0.07
**full sample**:			
molar amount *n*_0_ (mmol)	4.53 ± 0.01	6.11 ± 0.01	3.51 ± 0.01
density ρ (kg/m^3^)	1710 ± 2	1865 ± 1	1885 ± 2
velocity *c*_*l*_ (m/s)	1612.0 ± 5.3	3232.2 ± 1.3	2946.3 ± 1.3
modulus β (GPa)	4.44 ± 0.04	19.48 ± 0.03	16.36 ± 0.03
Δβ = β – β_0_ (GPa)	1.35 ± 0.05	1.00 ± 0.05	2.03 ± 0.10
Δβ/ϕ (GPa)	2.21 ± 0.09	2.87 ± 0.16	4.94 ± 0.26

a For details on the listed quantities and their determination please see text and Method section.

Experimentally, the elastic properties of adsorbates in nanoconfinement are usually determined with the aid of ultrasonic pulses.^18–20,23,25,38,40,41,44,45,51^ (Details on the experimental method can be found in the corresponding section at the end of this Letter.) Such an ultrasonic technique does not directly give access to the elasticity of the adsorbate, as only the effective moduli of the empty or filled porous material can be determined. However, the change of the effective modulus as a result of adsorption contains information on the modulus of the adsorbate itself that has to be extracted using an analysis. Consequently, for the experimental examination of the elastic properties of adsorbed argon in these samples we first need the elastic modulus of the empty sample. Thus, we have calculated the effective longitudinal modulus of the empty sample, $\beta_{0}=c_{l,0}^{2}\cdot \rho_{0}$ , using the measured velocity of a longitudinal ultrasonic pulse traveling through the empty sample, *c*_*l*,0_, and the effective density of the empty sample, ρ_0_ (see Table 1). Our measurements for the three samples yield a decrease of the modulus of the empty sample, β_0_, with increasing porosity ϕ. Indeed, the dependence of β_0_ on ϕ can be approximated by a linear function (see Figure 2b), i.e., it holds

1 $$\beta_{0}=\beta_{m,0}-a\cdot \phi$$

with the modulus of the matrix material, β_*m*,0_ (solid material with occluded porosity, the extrapolated value of $\beta_{0}\left(\phi =0\right)$ , cp. Figure 2b). The linear fit to our data for β_0_ yields a value of β_*m*,0_ = 38.97 ± 0.28 GPa. This value for β_*m*,0_ contrasts to the longitudinal modulus of quartz glass, β_*q*_ = 78.21 GPa,^68^ i.e., β_*m*,0_/β_*q*_ = 0.50. But such a strong difference between the modulus of the matrix material and pure quartz glass is typical for nanoporous glasses.^31,69^ Because of the observed nearly linear dependence between the modulus of the empty matrix and the porosity, we may assume that the elastic modulus of the matrix material, β_*m*_, is basically the same for all three samples $\left(\beta_{m}\left(\phi \right)=\beta_{m,0}\right)$ .

In three separate measurements, the three samples were filled with liquid argon at 86 K (i.e., near the normal boiling point of argon)^70^ via adsorption from the gas phase. The complete saturation of the pores (with the molar amount *n*_0_ of liquid argon) was reached by an increase of the vapor pressure up to the saturation vapor pressure of argon at 86 K. With the experimentally determined velocities of longitudinal pulses traveling through the filled samples, *c*_*l*_, and the density ρ of the filled samples, we thus have obtained the effective longitudinal moduli of the filled samples, $\beta =c_{l}^{2}\cdot \rho$ (see Table 1). As expected, in consequence of the filled pores these moduli β are higher than the moduli of the empty samples, β_0_. However, the measured contribution of the adsorbate to the effective longitudinal modulus, Δβ = β – β_0_, seems to show no systematic dependence on the porosity of the samples (see Figure 2d), though the mass of the adsorbed argon increases linearly with porosity (cp. Δρ = ρ – ρ_0_ in Figure 2c).

The measured effective moduli of a heterogeneous system depend, of course, on the moduli of its constituents (matrix material and adsorbate).^1,39,51,71^ Here we restrict ourselves to liquid adsorbates that exhibit no shear modulus, *G*_*Ar*,*ads*_ = 0. Owing to a general relation between bulk modulus, shear modulus, and longitudinal modulus, β_*Ar*,*ads*_ = *K*_*Ar*,*ads*_ + 4/3 · *G*_*Ar*,*ads*_, then β_*Ar*,*ads*_ = *K*_*Ar*,*ads*_ holds. In addition, the elastic properties depend on the microstructure and therefore there are various equations relating the modulus of the adsorbate to the measured effective moduli.^1,39,51,71^ All these make different assumptions and we cannot be sure a priori which one applies to our samples. Therefore, we proceed cautiously relying only on a few general features:

(i) In the high contrast limit (β_*Ar*,*ads*_ ≪ β_*m*_, *K*_*m*_) that applies for a liquid adsorbate in a solid matrix, a Taylor expansion up to the first order of Δβ = β – β_0_ at β_*Ar*,*ads*_ = 0 yields

2 $$\Delta \beta \propto \beta_{Ar,ads}$$

(ii) The modulus of a filled sample exceeds that of an unfilled one, β > β_0_. For our microstructures the measured modulus of the empty sample decreases linearly as a function of ϕ (see Figure 2b and eq 1). Therefore, we can assume a similar behavior for the filled samples, i.e.,

3 $$\beta =\beta_{m,0}-b\cdot \phi$$

with 0 < *b* < *a*, so that β > β_0_ (cp. eq 1).

Combining eqs 1–3 leads to

4 $$\frac{\Delta \beta }{\phi }\propto \beta_{Ar,ads}$$

A similar relation holds for the bulk modulus. A liquid adsorbate with *G*_*Ar*,*ads*_ = 0 does not alter the effective shear modulus of the empty matrix (*G* = *G*_0_). Then

$$\Delta K=K-K_{0}=\left(\beta -4/3\cdot G_{0}\right)-\left(\beta_{0}-4/3\cdot G_{0}\right)=\beta -\beta_{0}=\Delta \beta$$

holds. Together with β_*Ar*,*ads*_ = *K*_*Ar*,*ads*_ eq 4 then becomes

5 $$\frac{\Delta K}{\phi }\propto K_{Ar,ads}$$

Such a proportionality is predicted by several well-known effective medium formulas, e.g., the Voigt equation (see ref 72) or the Gassmann equation (the latter one for systems where *K*_0_ decreases linearly with ϕ, see ref 73). Since eq 1 is based on an experimental observation and eq 2 is a simple Taylor expansion, eq 3 as an analogy of eq 1 is the only assumption in the derivation of eqs 4 and 5.

According to eq 4 the contribution Δβ should increase linearly with the porosity, if β_*Ar*,*ads*_ is equal for all samples. However, as shown in Figure 2d, the measured data for Δβ seem to indicate no systematic dependence of the adsorbate’s contribution on ϕ. This obviously illustrates that the modulus of the adsorbed argon varies for the three samples with different pore sizes. In Figure 3a we have plotted Δβ/ϕ against the pore radius, *r*_*P*_. We observe that it increases considerably with decreasing pore radius, which reveals that the modulus of the adsorbed argon, β_*Ar*,*ads*_, increases with decreasing pore size. Actually, Figure 3b even exhibits a quasi linear dependence between the modulus of the adsorbate and the inverse pore size. This observation is—to our knowledge—the first experimental indication confirming the theoretical studies of Gor, Dobrzanski, and Maximov.^1,30,32,34,35,63^

**Figure 3:**
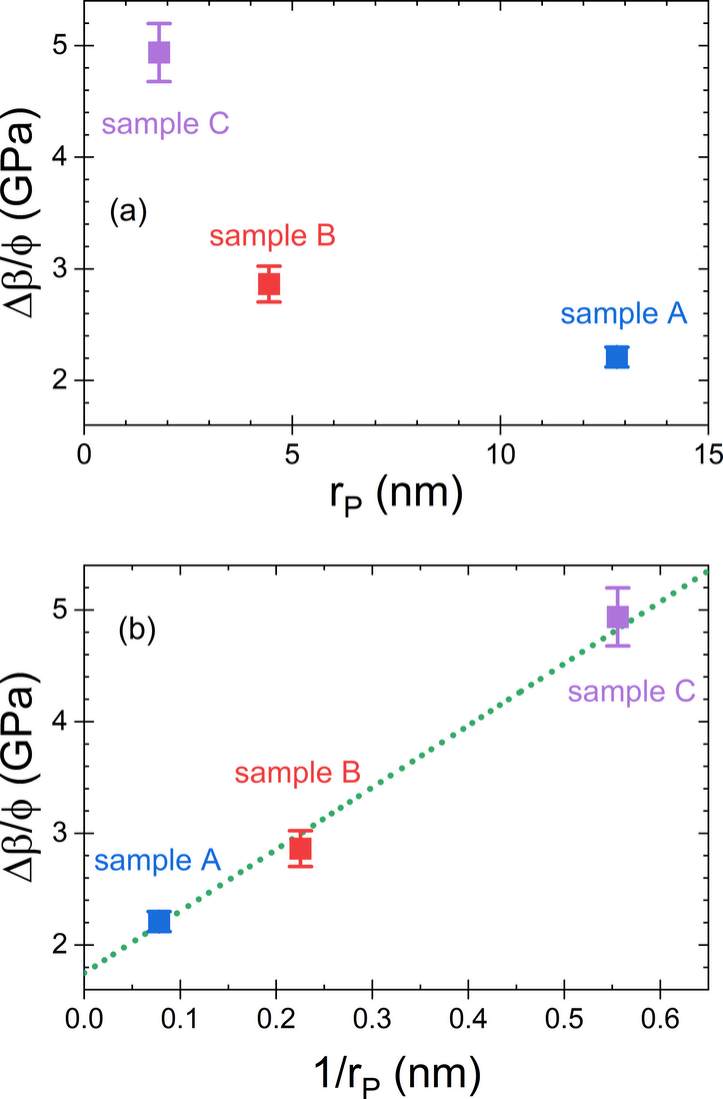
(a) Contribution of the adsorbate to the effective longitudinal modulus, Δβ = β – β_0_, scaled to the porosity ϕ as a function of the pore radius *r*_*P*_. As Δβ/ϕ is proportional to the modulus of the adsorbate, β_*Ar*,*ads*_ (see eq 4), the figure reveals an increase of the adsorbed argon’s longitudinal modulus with decreasing pore size. (b) The plot of Δβ/ϕ against 1/*r*_*p*_ indicates a linear relation between the two quantities. Thus, our measurements supply the first experimental indication of the theoretically predicted linear relation between the modulus of an adsorbate and the inverse pore size.^1,30,32,34,35,63^ The dotted line is a linear fit to the data.

Theoretical studies by the group of Gennady Y. Gor indicate that for argon in nanopores with a pore size above 10 nm the enhancement of the adsorbate’s modulus above the modulus of bulk argon is negligibly small.^30^ Thus, we can assume that the modulus of the adsorbed argon in sample A (*r*_*P*_ = 12.8 nm) is equal to the modulus of bulk argon, ${\left(\beta_{Ar,ads}\right)}_{A}=\beta_{Ar,bulk}$ . Consequently, we obtain

6 $$\frac{\beta_{Ar,ads}}{\beta_{Ar,bulk}}=\frac{\Delta \beta /\phi }{{\left(\Delta \beta /\phi \right)}_{A}}$$

according to eq 4, i.e., we can determine the ratio β_*Ar*,*ads*_/β_*Ar*,*bulk*_ simply by scaling the measured values displayed in Figure 3 by the value for sample A. The result is shown in Figure 4 as a function of the inverse pore radius revealing a linear increase of the modulus, i.e.,

7 $$\frac{\partial \beta_{Ar,ads}}{\partial \left(1/r_{P}\right)}=\mathrm{constant}$$

for adsorbed argon in nanopores that are smaller than in sample A (*r*_*P*_ ≲ 10 – 15 nm), in accordance with the theoretical prediction. Furthermore, Figure 4 shows that the impact of the pore size on the modulus of the adsorbed argon is significant: In our sample with the smallest pores (*r*_*P*_ = 1.8 nm) we find an enhancement of β_*Ar*,*ads*_ by a factor of more than two. This enhancement is somewhat stronger but roughly in the same range as that predicted by theory, which finds for spherical pores of similar sizes an enhancement by a factor of ≈1.4–1.7 (estimated using data at 87 K in refs 30 and 35 ). The deviation between the theoretically and experimentally determined absolute values for β_*Ar*,*ads*_/β_*Ar*,*bulk*_ may be attributed to differences in the pore geometry, the structure of the pore surfaces (which influences the interaction strength with the adsorbate) as well as to a not prefect linear dependence between β_0_ and ϕ, i.e., eq 1 is an approximation (cp. Figure 2b).

**Figure 4:**
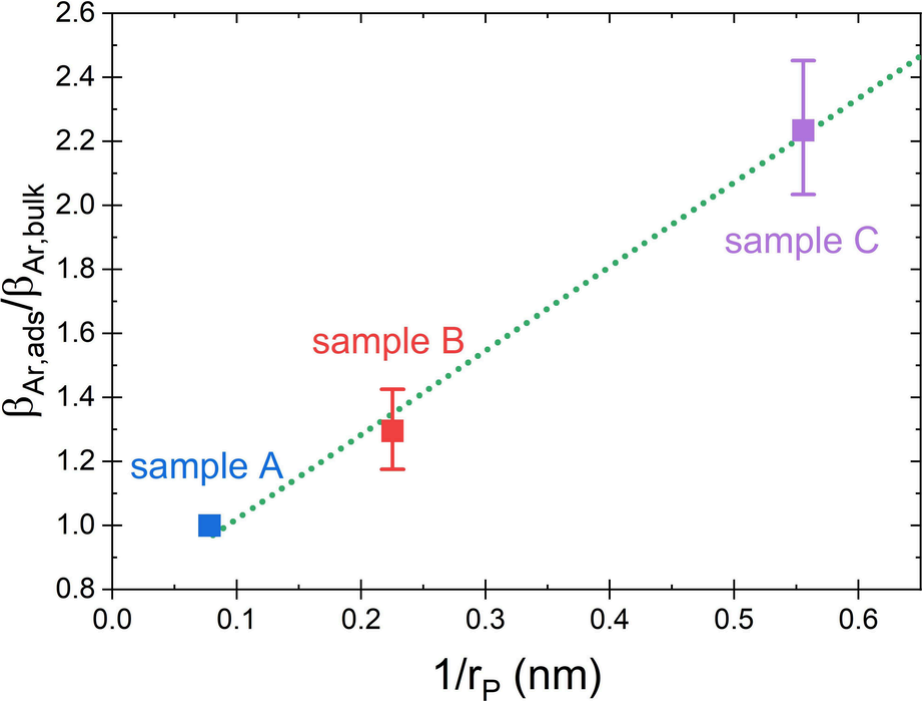
Longitudinal modulus β_*Ar*,*ads*_ of adsorbed argon in nanoporous glass scaled to the modulus of bulk argon, β_*Ar*,*bulk*_, as a function of the inverse pore radius, 1/*r*_*p*_. The data are calculated from our ultrasonic measurements assuming that the modulus in the largest pores (*r*_*P*_ = 12.8 nm, sample A) is equal to the modulus of bulk argon (assumption in accordance with theory). The observed linear relation between β_*Ar*,*ads*_/β_*Ar*,*bulk*_ and 1/*r*_*P*_ reveals a significant pore size dependence of β_*Ar*,*ads*_ (for pores that are smaller than in sample A). For the adsorbate in the smallest pores studied β_*Ar*,*ads*_ is more than twice as high as β_*Ar*,*bulk*_. The dotted line is a linear fit to the data.

In summary, we have shown with our ultrasonic measurements that the longitudinal modulus of adsorbed argon in nanopores of porous glasses increases almost linearly with the inverse pore size. Thus, our study represent – to our knowledge – the first experimental indications confirming this theoretically predicted pore size dependence of an adsorbate’s modulus. Assuming that in the largest pores (*r*_*P*_ = 12.8 nm) the adsorbate’s modulus is about bulk-like, in the smallest pores (*r*_*P*_ = 1.8 nm) the modulus of the confined argon is more than twice as high as that of bulk argon. Thus, even for argon, which exhibits only a weak interaction with the pore surface,^18,74^ we observe a considerable enhancement of the elastic modulus in small nanopores. Further experiments and theoretical studies are needed to reveal the influence of the interaction strength between adsorbate and pore surface on the pore size dependence of the modulus of the adsorbate. The experimentally observed effect of the pore size on the elastic modulus may in future be exploited for the development of new nanostructured materials with tailored elastic properties, for the study of the wave propagation in porous media (e.g., porous rocks containing nanopores), and also for the prediction of the impact of adsorption on the elasticity of porous storage materials.

## Method

For this experimental study we have used ultrasonic measurements. The ultrasonic signal was generated by applying voltage pulses to a piezoelectric crystal (LiNbO_3_, 36° Y-cut) that was glued with a silver epoxy (E-Solder 3021, Von Roll USA, Inc.) on the top surface of the sample. The generated ultrasonic pulses travel through the cylindrical porous samples (height *h* ≈ 0.2 – 0.5 cm, diameter *d* ≈ 0.9 – 1.1 cm), are reflected at its bottom, and then return to the piezoelectric crystal. The transit times of the generated longitudinal ultrasonic pulses for a round-trip through the empty or filled sample were measured with a digital oscilloscope. Using the measured transit times we have determined the ultrasonic velocities for the empty and filled samples (*c*_*l*,0_ and *c*_*l*_) and the effective longitudinal moduli $(\beta_{0}=c_{l,0}^{2}\rho_{0}$ and $\beta =c_{l}^{2}\rho )$ with the aid of the effective densities of the samples (ρ_0_ and ρ).

The sample was filled via adsorption of argon from the gas phase at 86 K (5.0 purity gas). The complete filling of the pores was reached by increasing the pressure in the sample cell up to the saturation vapor pressure of bulk argon. For this a gas distribution system is connected to the sealed copper sample cell that is mounted on the cold-head of a closed cycle helium cryostat. With the known volumes of gas distribution system and sample cell and the temperatures of both we have determined the amount of adsorbate, *n*_0_, using the ideal gas equation. Then, we have calculated the effective density of the filled sample using the mass *m*_*ads*_ = *n*_0_ · *M*_*Ar*_ of the experimentally determined molar amount of adsorbate, *n*_0_ (with the molar mass of argon, *M*_*Ar*_). The porosity ϕ = *V*_*pores*_/*V*_*sample*_ was determined using the volume $V_{pores}=V_{Ar,ads}\left(p_{0}\right)=n_{0}\cdot V_{M,Ar}$ of the adsorbate at *p*_0_ (with the molar volume of bulk argon at 86 K, *V*_*M*,*Ar*_).^70^ Before the start of each measurement the sample (in the sample cell) was evacuated at 300 K. Because of the cold-head higher temperatures were not possible. Therefore, we can assume that the pore surface remained covered with silanol OH groups.^67,69^

We have determined the pore size distribution of the samples from the desorption branches of isothermal argon sorption measurements at 86 K. The calculated pore radius *r*_*P*_ of each sample is the sum of the radius of withdrawing menisci, *r*_*K*_, and the thickness *t* of the argon layer remaining on the pore surface (i.e., we used the well-known Kelvin and Halsey equations that relate the relative pressure to *r*_*K*_ and *t*).^38,75^

More details on our method and the experimental setup can, for example, be found in the Supporting Information of ref 38.
